# Supplementary material for: The microbiota elicits compensatory adaptation in a seasonally-adapting animal host
Source: bioRxiv. 2025 Oct 14:2025.10.14.682128. Preprint. [Version 1] doi: 10.1101/2025.10.14.682128 (PMC12633038; doi:10.1101/2025.10.14.682128)
Supplement: Supplement 2 [file NIHPP2025.10.14.682128v1-supplement-2.pdf]

bioRxiv preprint doi: https://doi.org/10.1101/2025.10.14.682128; this version posted October 14, 2025. The copyright holder for this preprint (which was not certified by peer review) is the author/funder, who has granted bioRxiv a license to display the preprint in perpetuity. It is made available under aCC-BY 4.0 International license.

Supporting Figures

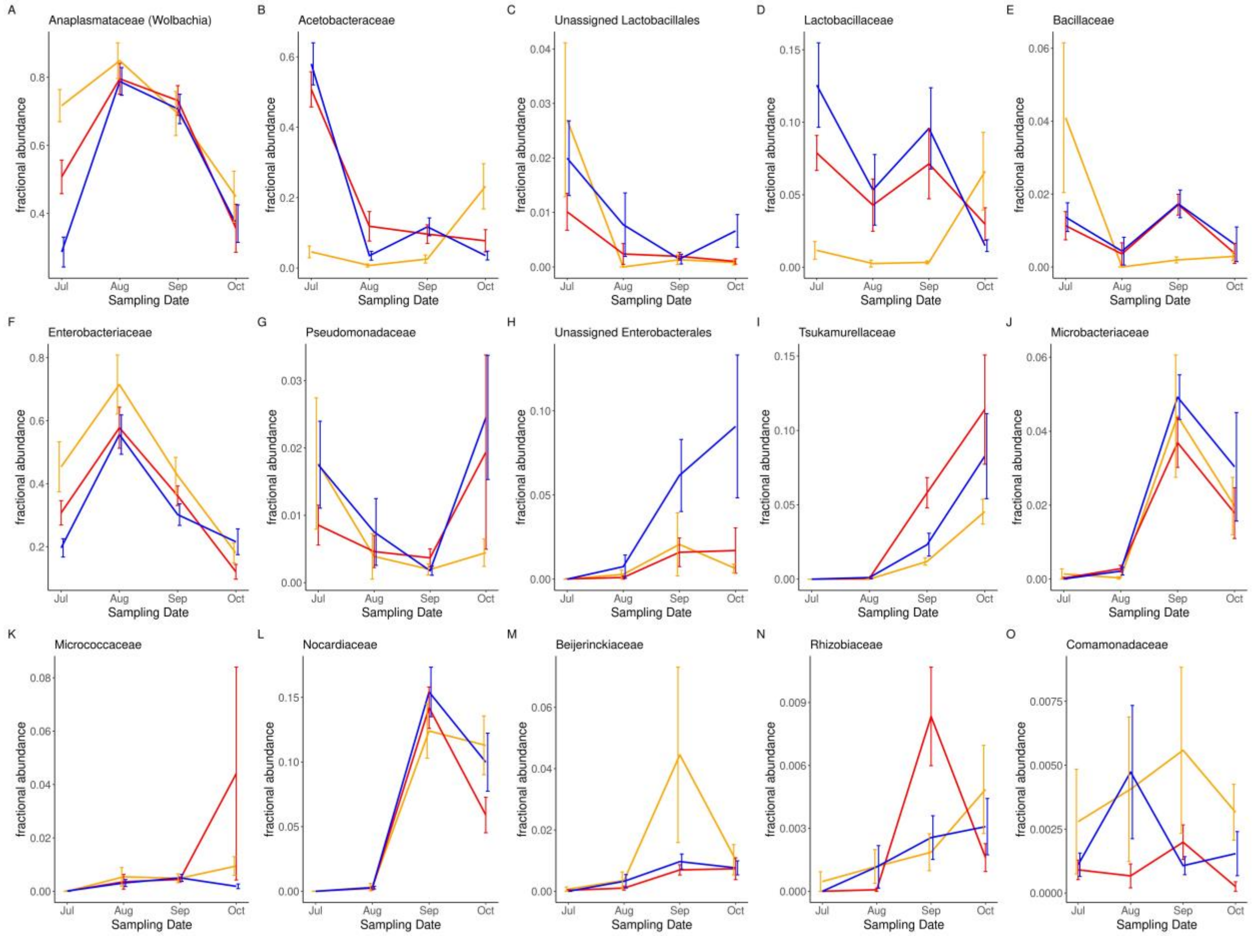

Fig S1. Differentially abundant ASVs. ASVs that significantly varied with time were identified using ANCOM. Analyses for *Anaplasmataceae* (*Wolbachia*) (A) was performed using an OTU table where *Wolbachia* reads were included and which was rarefied to 1000 reads per sample (**DATA S1**). All other taxa were identified from an OTU table where *Wolbachia* reads were exluded and rarefied to 537 reads per sample (**DATA S2**). ASVs in D) and H) also varied significantly with the microbial treatment. Line colors represent mesocosms inoculated with *Ao* (red), *As* (orange), or *Wp* (blue).

bioRxiv preprint doi: https://doi.org/10.1101/2025.10.14.682128; this version posted October 14, 2025. The copyright holder for this preprint (which was not certified by peer review) is the author/funder, who has granted bioRxiv a license to display the preprint in perpetuity. It is made available under aCC-BY 4.0 International license.

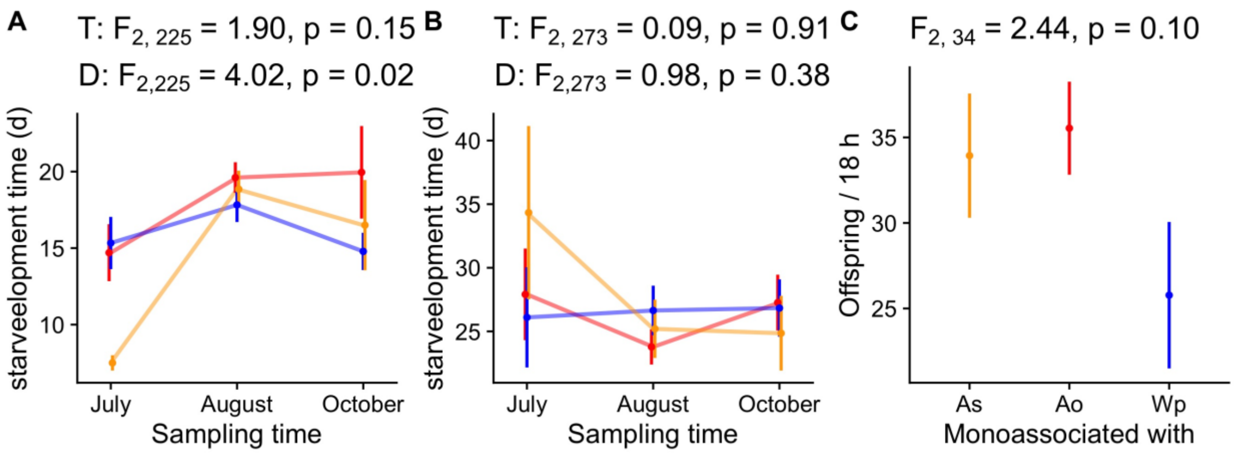

Fig S2 Fecundity of seasonally-selected *D. melanogaster*. The number of offspring produced by a single female in 18 hours was measured in A) axenic or B) gnotobiotic flies taken from the field and reared in the laboratory after several generations of common garden, or C) F3 offspring of the starting populations in the cages, monoassociated with the treatment strains in the laboratory.

bioRxiv preprint doi: https://doi.org/10.1101/2025.10.14.682128; this version posted October 14, 2025. The copyright holder for this preprint (which was not certified by peer review) is the author/funder, who has granted bioRxiv a license to display the preprint in perpetuity. It is made available under aCC-BY 4.0 International license.

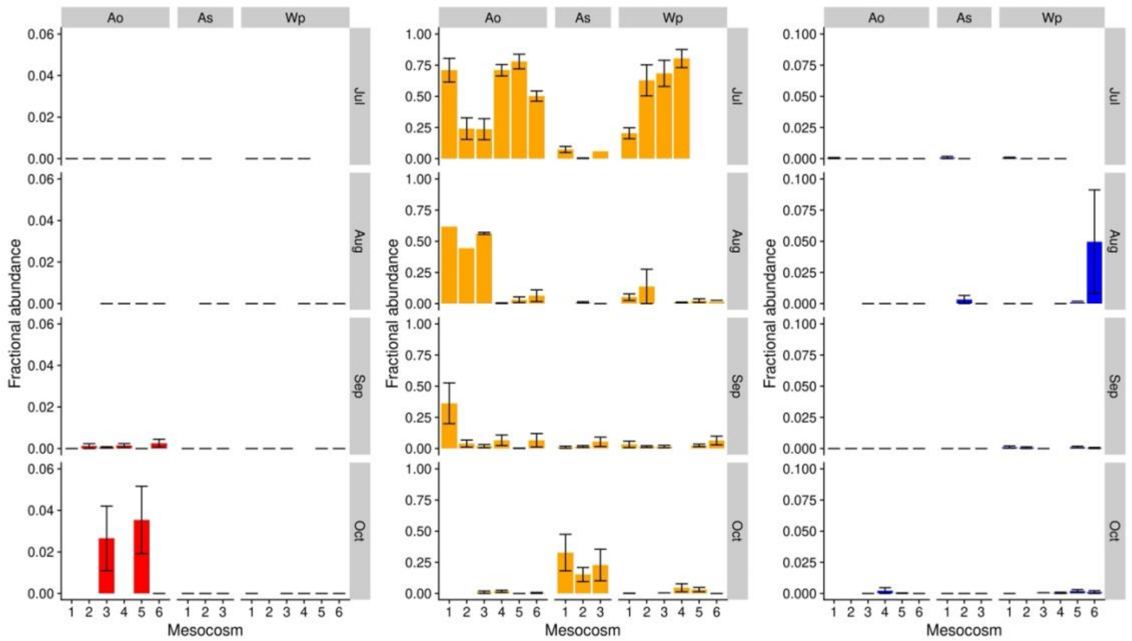

Fig S3. Detection of inoculated strains throughout the experiment. Flies were inoculated with *Ao*, *As*, or *Wp* over the course of four months (Jul-Oct). Separate panels for the fractional abundance of *Ao* (red), *As* (orange), and *Wp* (blue) show the presence of ASVs with full-length 100% matches to the inoculated treatment strains.

bioRxiv preprint doi: https://doi.org/10.1101/2025.10.14.682128; this version posted October 14, 2025. The copyright holder for this preprint (which was not certified by peer review) is the author/funder, who has granted bioRxiv a license to display the preprint in perpetuity. It is made available under aCC-BY 4.0 International license.

Table S1. PERMANOVA results

		Bray-Curtis					Unweighted Unifrac					Weighted Unifrac			
	df	SS	R^2	F	p		SS	R^2	F	p		SS	R^2	F	p
Bacteria	2	1.66	0.02	3.78	$<10^{-3}$		0.73	0.02	3.01	$<10^{-3}$		0.44	0.02	3.52	$<10^{-3}$
Date	3	18.95	0.2	28.69	$<10^{-3}$		8.21	0.17	22.51	$<10^{-3}$		6.26	0.23	33.43	$<10^{-3}$
Bacteria * Date	6	4.23	0.05	3.21	$<10^{-3}$		1.09	0.02	1.49	0.01		1.44	0.05	3.84	$<10^{-3}$
Residual	305	67.13	0.72				37.08	0.78				19.02	0.69		
Total	317	93.3	1				47.4	1				27.65	1		
